# Integrating postal HIV testing into the HIV care cascade in Japan: a public health centre model

**DOI:** 10.1002/jia2.70086

**Published:** 2026-02-15

**Authors:** Kota Iwahashi, Keisuke Ejima, Naho Tsuchiya, Nittaya Phanuphak, Akifumi Imamura

**Affiliations:** ^1^ Community Centre AKTA Tokyo Japan; ^2^ Lee Kong Chian School of Medicine Nanyang Technological University Singapore Singapore; ^3^ National Centre for Infectious Diseases Singapore Singapore; ^4^ Yamato Home Medical Care Clinic Kurihara Miyagi Japan; ^5^ Tohoku Medical Megabank Organization Tohoku University Miyagi Japan; ^6^ Institute of HIV Research and Innovation (IHRI) Bangkok Thailand; ^7^ Department of Infectious Diseases Tokyo Metropolitan Cancer and Infectious Diseases Centre Komagome Hospital Tokyo Japan

1

HIV testing is delivered through three principal modalities: facility‐based testing; remote self‐sampling/postal testing (samples mailed to a laboratory); and self‐testing (HIVST). By July 2024, 107 countries had policies supporting HIVST, of which 71 reported routine implementation, while the remaining 38 had supportive policies but had not yet reported routine implementation [1, 2, 3]. In Japan, however, HIVST for at‐home use has not yet been approved by the government, partly due to concerns about follow‐up and linkage to care after users had reactive results.

Japan's HIV epidemic remains concentrated among men who have sex with men (MSM): in 2023, 71% of new HIV acquisitions were attributed to male‐to‐male sexual contact [4]. Accordingly, MSM‐focused interventions are pivotal for prevention and case‐finding, and close collaboration between public health services and community‐based organizations (CBOs)—particularly those serving lesbian, gay, bisexual, transgender and queer (LGBTQ) communities—is essential to expand access, provide accurate information about testing options and reduce stigma.

Within this context, Public Health Centres (PHCs) have long anchored Japan's HIV response. They are widely established nationwide and, as publicly funded institutions operated by local governments with national subsidies, offer free, anonymous HIV testing, pre‐ and post‐test counselling, and referral to care (Figure 1) [4]. In 2023, municipalities conducted 106,137 HIV tests and provided 86,088 consultations through PHCs; 316 people screened positive, which represents one‐third of the 983 people with newly reported HIV infections nationwide that year. PHCs have also worked with CBOs across the country to widen access to their services, including PHC‐led collaborations in which CBOs support community outreach, testing promotion at LGBTQ venues and events, training of PHC staff, and navigation from community‐based activities to PHC‐provided HIV testing and follow‐up [5].

**Figure 1:**
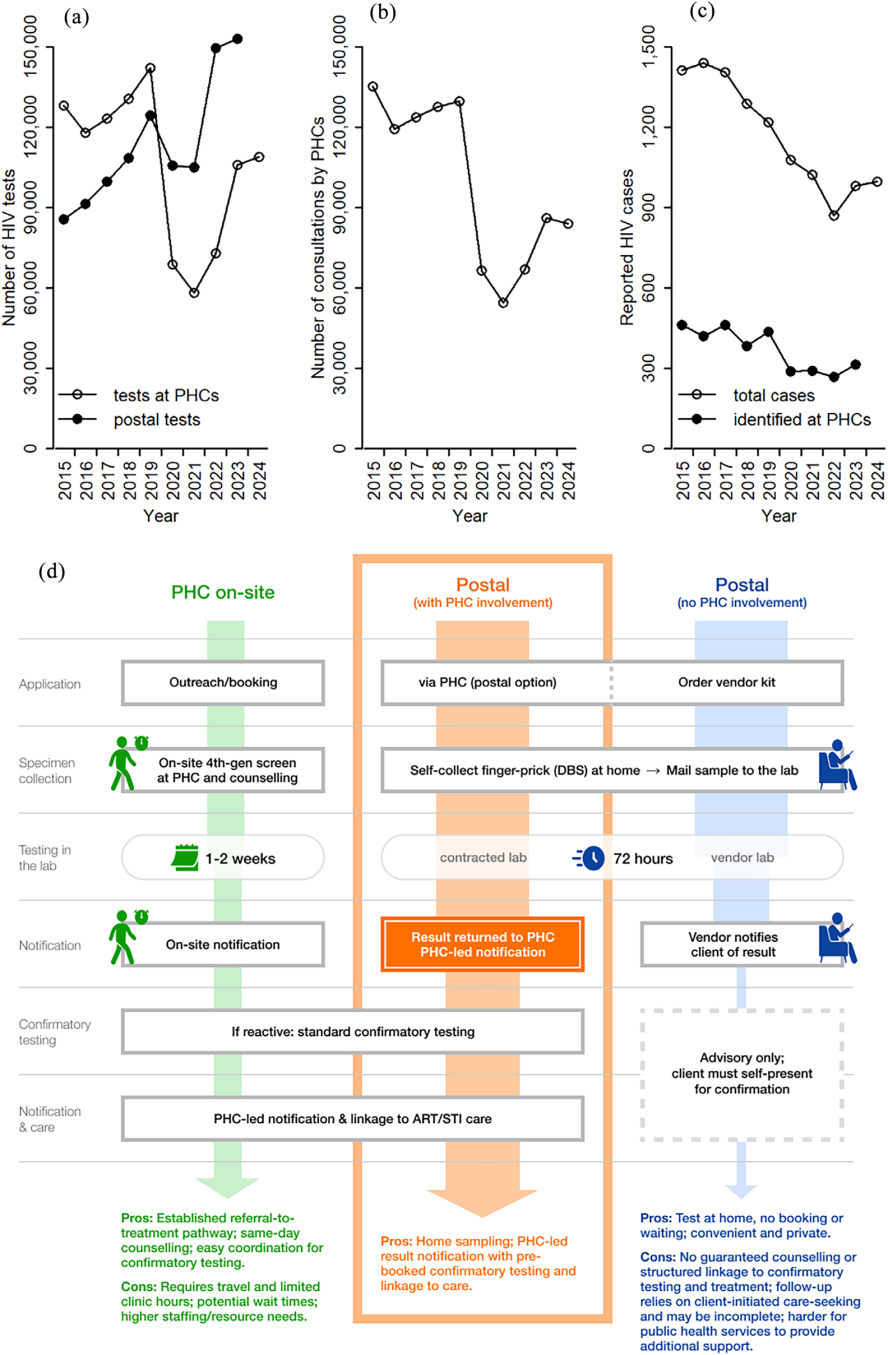
HIV testing, counselling and case ascertainment at Public Health Centres and HIV postal tests, Japan, 2015–2024. (a) Number of HIV tests conducted by PHCs and HIV postal tests; (b) number of counselling sessions provided by PHCs; (c) newly reported HIV diagnoses (HIV acquisitions and AIDS diagnoses combined) and those that were first identified by PHCs; (d) HIV testing cascade in Japan: PHC on‐site testing, postal testing (no PHC involvement) and postal testing (with PHC involvement). Abbreviations: 4th‐gen, fourth‐generation HIV test; ART, antiretroviral therapy; DBS, dried blood spot; PHC, public health centre; STI, sexually transmitted infection.

Since the early 2000s, access to and uptake of postal HIV testing has expanded in Japan [6]. In recent years, CBOs and their clinical/academic partners started pilot projects to extend the reach of postal testing among MSM and evaluate the feasibility and acceptability of self‐sampling using finger‐prick and dried blood spots (DBS; e.g. the HIVcheck programme [7]). Japan's Ministry of Health, Labor and Welfare research group has surveyed postal testing vendors annually since 2005, quantifying testing volumes, HIV positivity and linkage indicators [6]. In 2023, vendors reported 153,037 postal tests—figures that exceeded PHC‐site testing volumes that year [6].

The contrast in testing cascades helps explain such dynamics: PHC‐led testing is provider‐delivered and labour‐intensive, whereas the postal testing pathway offers more limited services than PHCs (Figure 1). The self‐test process offers privacy, convenience and access after hours. However, there are some issues with postal testing as well. In 2023, of the 124 reactive results identified through postal testing, only 33% of those tested were referred to medical facilities for follow‐up, and attendance was confirmed for just 16% of those with reactive results, highlighting substantial gaps in linkage to care [6].

In June 2025, the Japanese government issued *Guidelines for HIV Postal Testing Conducted by Public Health Centres and Related Institutions* [8], in which postal testing was formally positioned as a PHC service option and explicitly defined as pre‐screening. Reactive or indeterminate results must proceed through the standard two‐stage algorithm, with PHC‐led result notification and linkage (Figure 1). PHCs may contract vetted vendors, specify kit contents and data returns, and pre‐book confirmatory appointments, bringing the postal cascade under PHC accountability. However, these guidelines are non‐binding, and implementation and operational workflows remain at the discretion of each PHC; actual practices are expected to vary across jurisdictions.

Despite growing international evidence supporting diversified HIV testing modalities, discussions around postal testing in Japan have often been shaped by concerns about potential harms (e.g. delayed linkage to care, loss of counselling opportunities and misuse of test kits) in the absence of systematic evaluation of these risks against observed outcomes. While precaution is essential in public health policy, reliance on assumed risks may inadvertently slow the integration of testing approaches that improve access for populations less likely to use facility‐based services. This highlights the need for policies grounded in comparative, outcome‐based evidence.

From the community perspective, we generally welcome the government's official approval to use postal HIV testing as part of PHC services. At the same time, we are concerned that the long‐standing roles of PHCs may risk being undermined. Postal HIV testing conducted under PHC auspices should complement and not substitute on‐site PHC services. As representatives and partners of local CBOs, we urge the Japanese government to take clear responsibility in setting a coherent direction for inclusion of postal HIV testing in the national public health programme, in close partnership with relevant communities. Specifically, we recommend:

- **Integration of HIV prevention as well as treatment services**: Link pre‐exposure and post‐exposure prophylaxis (PrEP and PEP) services with self‐sampling and postal testing to ensure seamless connections to prevention, confirmatory testing and treatment services.
- **Establishment of clear referral pathways between communities and PHCs**: Formal collaborations should be encouraged between community‐led and community‐based organizations and PHCs to jointly generate demand for postal testing, provide virtual support for self‐sampling and facilitate navigation to PrEP, PEP, and confirmatory testing and treatment services.
- **Expanded testing choices**: Maintain a diverse range of HIV testing options so that individuals can make informed decisions that best fit their lifestyle and context, ensuring that no single option replaces another.
- **Evidence‐based policy decision‐making**: Support postal HIV testing—and HIVST when authorized—as part of a diversified testing portfolio, with policies grounded in empirical, outcome‐based evidence. In particular, prioritize prospective evaluations comparing (1) time to confirmatory diagnosis, treatment and linkage to care; (2) user acceptability and equity across populations differing by age, geography, socio‐economic status, sexual identity and prior engagement with PHC services; and (3) the cost‐effectiveness of each modality individually as well as mixed strategies (e.g. postal testing plus PHC‐based services).
- **Technology‐enabled linkage to care**: Design HIVST and postal testing programmes with embedded digital linkage mechanisms to reduce attrition after testing. For example, test kits can include QR codes or short links that connect users to verified clinical resources, such as online appointment systems for confirmatory testing and treatment or access to PrEP counselling and services. Such low‐burden digital tools can strengthen linkage to care while preserving user autonomy and privacy, and should be evaluated alongside testing uptake and clinical outcomes.

In summary, postal HIV testing represents an important opportunity to expand access to testing in Japan, particularly for populations less likely to engage with facility‐based services. However, its value to improving public health depends on deliberate integration within a PHC‐centred framework that safeguards timely linkage to confirmatory testing, treatment and prevention services. Rather than viewing postal testing as a substitute for established PHC functions, Japan should position it as a complementary modality within a diversified testing portfolio, guided by empirical evaluation and close collaboration with communities. Clear national direction, coupled with local flexibility and accountability, will be essential to ensure that innovations in HIV testing strengthen—rather than fragment—the HIV care cascade.

## COMPETING INTERESTS

All authors declare that they have no competing interests.

## AUTHORS’ CONTRIBUTIONS

Conceived and designed the study: KI, KE and AI. Obtained and analysed the data: KI and KE. Wrote the paper: KI and KE. Edited the paper: NT, NP and AI. All authors read and approved the final manuscript.

## FUNDING

The research was funded by Japan Science and Technology Agency (JST), PRESTO, Japan (JPMJPR23R3) (to KE).

## Data Availability

All data used to generate Figure 1 are available in references [4] and [6].
